# A Gentle Hint

**Published:** 1882-03

**Authors:** 


### A GENTLE HINT.

### TO ALL WHOM IT MAY CONCERN.

PUT YOURSELF IN OUR PLACE.—If our subscribers who are in arrears in their dues, will imagine themselves in our place for a moment, they will appreciate the position exactly, and will at once send their postal orders. What would be the estimation in which a physician would hold a patient who utterly disregarded for months the frequent reminder in the shape of a quarterly statement? Think on these things and govern yourself accordingly, for it is just as necessary for our printer to be paid as for your butcher!

We shall certainly stop sending the journal to such as are largely behind in payment, and place their account in a process of collection which will add costs!—N. Y. *Med. Times.*

